# Supplementary material for: Dosage Optimisation of Trimethoprim and Sulfamethoxazole for the Treatment of an Avian Pathogenic Strain of Escherichia coli in Broiler Chickens
Source: Antibiotics (Basel). 2023 Dec 20;13(1):11. doi: 10.3390/antibiotics13010011 (PMC10812532; doi:10.3390/antibiotics13010011)
Supplement: Supplementary file 1 [file antibiotics-13-00011-s001.zip › antibiotics-2746070-supplementary.pdf]

Supplementary File 1

Dosage Optimisation of Trimethoprim and Sulfamethoxazole for the Treatment of an Avian Pathogenic Strain of *Escherichia coli* in Broiler Chickens

Kamil Stastny [1], Nikola Hodkovicova [1], Martin Jerabek [2], Michal Petren [2], Michaela Viskova [2], Aneta Papouskova [3], Iva Bartejsova [1], Kristina Putecova-Tosnerova [1], Michaela Charvatova [1], Monika Zouharova [1], Katarina Matiaskova [1] and Katerina Nedbalcova [1,*]

[1] Veterinary Research Institute, Hudcova 296/70, 621 00 Brno, Czech Republic; kamil.stastny@vri.cz (K.S.); nikola.hodkovicova@vri.cz (N.H.); iva.bartejsova@vri.cz (I.B.); kristina.tosnerova@vri.cz (K.P.-T.); michaela.charvatova@vri.cz (M.C.); monika.zouharova@vri.cz (M.Z.); katarina.matiaskova@vri.cz (K.M.)

[2] Tekro, Ltd., Visnova 484/2, 140 00 Prague, Czech Republic; m.jerabek@tekro.cz (M.J.); m.petren@tekto.cz (M.P.); m.viskova@tekro.cz (M.V.)

[3] Institute of Infection Diseases and Microbiology, Faculty of Veterinary Medicine, University of Veterinary Sciences, Palackeho 1-3, 612 42 Brno, Czech Republic; papouskovaa@vfu.cz

* Correspondence: katerina.nedbalcova@vri.cz; Tel.: +42-0728943327

Abstract: Based on pharmacokinetic studies carried out according to the methodologies defined by the European Medicines Agency (EMA) using mass spectrometry analysis, a new formulation of a veterinary drug for the treatment of broiler chickens is proposed. Currently, the traditional trimethoprim–sulfamethoxazole drug used for broilers is applied in a 1:5 ratio, and the recommended dose is 45 mg kg^{-1} of live weight administered at 24 h intervals for 3 to 5 days. In this study, we propose a novel combination containing similar active substances in a newly established ratio of 1:4, with a recommended dosage of 20 mg kg^{-1} of live weight administered at 24 h intervals for 3 to 5 days. With this method, the currently recommended dose of the traditional trimethoprim–sulfamethoxazole drug used for broilers can be reduced by more than half. The efficacy of the newly designed formulation and dosage of the drug was verified in a bioassay for the treatment of broilers experimentally infected with an avian pathogenic strain of *Escherichia coli*. In the experiment, we compared the newly designed dosage with the traditional dosage in terms of efficacy and dosage. There were no statistically significant differences between the two drugs in efficacy regarding the survival of chickens after experimental infection or changes in their health status. The experimental results suggest that a significant reduction in the recommended daily dose of drugs containing trimethoprim and sulfamethoxazole for the treatment of bacterial infections in broilers is possible and can support the prudent use of antimicrobials, including the limitation of their overuse.

Keywords: poultry; *Escherichia coli*; APEC; antimicrobials; LC-MS/MS; pharmacokinetics

Table S1. Body weight (g).

		Day of Experiment							
**Groupe**	**Animals**	19	20	21	22	23	24	25	26
I.	1	700	773	840	873	984	1070	1100	1222
	2	847	945	1005	1088	1135	1254	1304	1394
	3	682	712	755	829	903	966	1046	1130
	4	763	852	924	1003	1088	1181	1195	1311
	5	677	729	785	856	929	980	991	+
	6	702	772	836	916	999	1067	1138	1256
	7	741	833	899	1021	1054	1179	1191	1308
	8	793	872	926	1008	1068	1150	1185	1292
	9	841	918	975	1082	1127	1191	1255	1282
	10	705	774	837	910	980	1060	1095	1211
Average		*745.1*	*818*	*878.2*	*958.6*	*1026.7*	*1109.8*	*1150*	*1267.3*
SD		*63.5*	*78.6*	*81.0*	*93.8*	*80.0*	*95.6*	*95.1*	*74.7*
CI (95%)		*699.6;790.6*	*761.7;874.2*	*820.2;936.2*	*891.5;1025.7*	*969.5;1083.9*	*1041.4;1178.2*	*1082;1218*	*1209.9;1324.7*

**Groupe**	**Animals**	19	20	21	22	23	24	25	26
II.	11	627	697	750	815	911	956	973	1060
	12	687	864	853	939	1021	1120	1133	+
	13	808	895	958	1027	1067	1199	1222	1330
	14	719	789	840	901	941	1001	1040	1125
	15	854	950	1021	1074	1132	1218	1265	1360
	16	667	726	780	846	896	986	1029	1133
	17	700	767	796	873	906	1048	1104	1125
	18	897	992	1058	1138	1202	1311	1373	1492
	19	805	855	897	946	981	1044	1088	1200
	20	721	771	809	853	860	931	966	+

Average		*748.5*	*830.6*	*876.2*	*941.2*	*991.7*	*1081.4*	*1119.3*	*1228.1*
SD		*87.6*	*96.8*	*104.9*	*106.9*	*112.3*	*126.1*	*132.0*	*149.7*
CI (95%)		*685.8;811.2*	*761.4;899.8*	*801.1;951.3*	*864.7;1017.7*	*911.4;1072.1*	*991.2;1171.6*	*1024.9;1213.7*	*1103;1353.2*

**Groupe**	**Animals**	19	20	21	22	23	24	25	26
III.	31	675	632	+	+	+	+	+	+
	32	635	612	+	+	+	+	+	+
	33	683	+	+	+	+	+	+	+
	34	642	624	+	+	+	+	+	+
	35	660	654	+	+	+	+	+	+
	36	670	576	+	+	+	+	+	+
	37	668	646	+	+	+	+	+	+
	38	615	533	+	+	+	+	+	+
	39	740	+	+	+	+	+	+	+
	40	651	635	+	+	+	+	+	+
Average		*663.9*	*614*						
SD		*33.7*	*40.5*						
CI (95%)		*639.8;688*	*580.1;647.9*						

**Groupe**	**Animals**	19	20	21	22	23	24	25	26
IV.	21	827	853	890	939	1012	1087	1163	1216
	22	830	869	910	969	1070	1144	1172	1237
	23	828	894	915	973	1074	1116	1190	1292
	24	777	853	860	938	1114	1153	1183	1287
	25	766	835	858	909	985	1050	1134	1180
	26	737	796	821	878	1006	1036	1130	1213
	27	726	786	794	877	978	1054	1062	1230
	28	888	957	973	1127	1259	1306	1370	1516
	29	856	932	935	994	1090	1159	1236	1345

30	804	870	893	973	1071	1148	1260	1340
Average	*803.9*	*864.5*	*884.9*	*957.7*	*1065.9*	*1125.3*	*1190*	*1285.6*
SD	*51.9*	*53.8*	*53.3*	*71.9*	*82.4*	*78.7*	*84.1*	*97.9*
CI (95%)	*766.7;841.1*	*826;903*	*846.8;923*	*906.3;1019.1*	*1007;1124.8*	*1069;1181.6*	*1129.8;1250.2*	*1215.5;1355.7*

Table S2. Average daily grain - ADR (g).

		Day of Experiment						
**Groupe**	**Animals**	20	21	22	23	24	25	26
I.	1	73	67	33	111	86	30	122
	2	98	60	83	47	119	50	90
	3	30	43	74	74	63	80	84
	4	89	72	79	85	93	14	116
	5	52	56	71	73	51	11	+
	6	70	64	80	83	68	71	118
	7	92	66	122	33	125	12	117
	8	79	54	82	60	82	35	107
	9	77	57	107	45	64	64	27
	10	69	63	73	70	80	35	116
Average		*72.9*	*60.2*	*80.4*	*68.1*	*83.1*	*40.2*	*99.7*
SD		*20.0*	*8.2*	*23.3*	*22.8*	*24.0*	*25.1*	*30.3*
CI (95%)		*58.6;87.2*	*54.3;66.1*	*63.7;97.1*	*51.8;84.4*	*65.9;100.3*	*22.2;58.2*	*76.4;123*
**Groupe**	**Animals**	20	21	22	23	24	25	26
II.	11	70	53	65	96	45	17	87
	12	177	-11	86	82	99	13	+
	13	87	63	69	40	132	23	108
	14	70	51	61	40	60	39	85

	15	96	71	53	58	86	47	95
	16	59	54	66	50	90	43	104
	17	67	29	77	33	142	56	21
	18	95	66	80	64	109	62	119
	19	50	42	49	35	63	44	112
	20	50	38	44	7	71	35	+
Average		*82.1*	*45.6*	*65*	*50.5*	*89.7*	*37.9*	*91.4*
SD		*37.3*	*23.7*	*13.7*	*25.7*	*31.5*	*16.1*	*30.8*
CI								
(95%)		*55.4;108.8*	*28.6;62.6*	*55.2;74.8*	*32.1;68.9*	*67.2;112.2*	*26.4;49.4*	*65.6;117.1*
**Groupe**	**Animals**	20	21	22	23	24	25	26
	31	-43	+	+	+	+	+	+
	32	-23	+	+	+	+	+	+
	33	+	+	+	+	+	+	+
	34	-18	+	+	+	+	+	+
	35	-6	+	+	+	+	+	+
III.	36	-94	+	+	+	+	+	+
	37	-22	+	+	+	+	+	+
	38	-82	+	+	+	+	+	+
	39	+	+	+	+	+	+	+
	40	-16	+	+	+	+	+	+
Average		*-38*						
SD		*32.7*						
CI								
(95%)		-						
**Groupe**	**Animals**	20	21	22	23	24	25	26
	21	26	37	49	73	75	76	53
IV.	22	39	41	59	101	74	28	65
	23	66	21	58	101	42	74	102

24	76	7	78	176	39	30	104
25	69	23	51	76	65	84	46
26	59	25	57	128	30	94	83
27	60	8	83	101	76	8	168
28	69	16	154	132	47	64	146
29	76	3	59	96	69	77	109
30	66	23	80	98	77	112	80
Average	*60.6*	*20.4*	*72.8*	*108.2*	*59.4*	*64.7*	*95.6*
SD	*16.1*	*12.4*	*31.0*	*30.3*	*18.0*	*32.7*	*39.0*
CI (95%)	*49.1;72.1*	*11.5;29.3*	*50.6;95*	*86.6;129.8*	*46.6;72.2*	*41.3;88.1*	*67.7;123.5*

(+ = death)
